# AT2R agonist NP‐6A4 mitigates aortic stiffness and proteolytic activity in mouse model of aneurysm

**DOI:** 10.1111/jcmm.15342

**Published:** 2020-05-18

**Authors:** Neekun Sharma, Anthony M. Belenchia, Ryan Toedebusch, Lakshmi Pulakat, Chetan P. Hans

**Affiliations:** ^1^ Department of Cardiovascular Medicine University of Missouri Columbia MO USA; ^2^ Dalton Cardiovascular Research Center University of Missouri Columbia MO USA; ^3^ Medical Pharmacology and Physiology University of Missouri Columbia MO USA; ^4^ Molecular Cardiology Research Institute Department of Medicine Tufts Medical Center Tufts University School of Medicine Boston MA USA

**Keywords:** abdominal aortic aneurysm, AT2R, endothelial dysfunction, NP‐6A4

## Abstract

Clinical and experimental studies show that angiotensin II (AngII) promotes vascular pathology via activation of AngII type 1 receptors (AT1Rs). We recently reported that NP‐6A4, a selective peptide agonist for AngII type 2 receptor (AT2R), exerts protective effects on human vascular cells subjected to serum starvation or doxorubicin exposure. In this study, we investigated whether NP‐6A4–induced AT2R activation could mitigate AngII‐induced abdominal aortic aneurism (AAA) using AngII‐treated *Apoe^−/−^* mice. Male *Apoe^−/−^* mice were infused with AngII (1 µg/kg/min) by implanting osmotic pumps subcutaneously for 28 days. A subset of mice was pre‐treated subcutaneously with NP‐6A4 (2.5 mg/kg/day) or vehicle for 14 days prior to AngII, and treatments were continued for 28 days. NP‐6A4 significantly reduced aortic stiffness of the abdominal aorta induced by AngII as determined by ultrasound functional analyses and histochemical analyses. NP‐6A4 also increased nitric oxide bioavailability in aortic tissues and suppressed AngII‐induced increases in monocyte chemotactic protein‐1, osteopontin and proteolytic activity of the aorta. However, NP‐6A4 did not affect maximal intraluminal aortic diameter or AAA incidences significantly. These data suggest that the effects of AT2R agonist on vascular pathologies are selective, affecting the aortic stiffness and proteolytic activity without affecting the size of AAA.

## 1 INTRODUCTION

Cardiovascular diseases are the leading cause of death worldwide. Decades of research has established that the components of renin‐angiotensin system (RAS) play a central role in maintaining cardiovascular physiology, including arterial compliance and regulation of blood pressure.^1^, ^2^ However, overactivation of RAS caused by metabolic imbalances such as obesity, hyperlipidaemia and diabetes, or infection results in increased presence of angiotensin II (AngII) and up‐regulation of AngII type 1 receptor (AT1R). Hyperactive AT1R induces inflammatory signalling resulting in oxidative stress, and pathological sequelae of vasculopathies that cause hypertension, and micro‐ and macrovascular damage including abdominal aortic aneurysm (AAA).^3^, ^4^

It is well established that AngII also has a vascular protective role that is mediated via its type 2 receptor, AT2R.^5^, ^6^ AT2R, encoded by the X‐linked *Agtr2* gene, is a G protein‐coupled receptor and shares only 34% homology with AT1R.^7^ AT2R expression is localized to vascular endothelial cells (ECs), vascular smooth muscle cells (vSMCs) and on the cells of inflammatory and immune origins such as monocytes and T cells.^8^, ^9^, ^10^ AT2R is a reparative molecule, and its activation enhances microvascular perfusion and oxygenation and regeneration of muscle.^11^, ^12^

NP‐6A4 is a patented peptide AT2R agonist (Novopyxis Inc, Boston, MA), which has acquired FDA's Orphan Drug designation for the treatment of paediatric cardiomyopathy.^8^ We have recently reported that AT2R up‐regulation with NP‐6A4 activates protective signalling in human ECs by increasing endothelial nitric oxide synthase (eNOS) expression and bioavailability of nitric oxide (NO) and in vSMCs via suppression of reactive oxygen species.^8^ Similarly, we have shown that NP‐6A4 protects mouse HL‐1 cardiomyocytes and human coronary artery vSMCs from acute nutrient deficiency.^13^ These protective effects of AT2R stimulation have been well supported by other evidence for maintaining vascular integrity of the aorta.^14^, ^15^

The contribution of AT2R stimulation in abdominal aortic pathologies remains controversial. In an experimental elastase‐induced AAA model in rat, AT2R stimulation with C21, a small molecule agonist of the AT2R, prevented aneurysm progression by preserving the functional and structural morphology of aorta.^14^ However, in a mouse model, AT2R deficiency had no effects on AngII‐induced AAA and atherosclerosis.^16^ These conflicting reports warrant further studies to confirm the roles of AT2R stimulation in the settings of AngII‐induced aortic pathologies.

The literature shows that in male *Apoe^−/−^* mice, treatment with AngII (1 µg/kg/min) results in AAA.^17^ Since mouse vasculature expresses both AT1R and AT2R, the effect of AT1R seems to override any protective effects of AT2R in this model. To date, no studies have examined the effects of AT2R activation in the presence of high AngII concentrations in the context of vasculopathies including AAA. Therefore, in the present study, we aimed to determine whether activation of AT2R by NP‐6A4 modulates AngII‐induced vasculopathies in *Apoe^−/−^* mice. Our results show that NP‐6A4–induced AT2R signalling attenuates AngII‐induced aortic stiffness as a primary outcome. This protective effect involved increase in the bioavailability of NO in ECs and suppression of osteopontin and matrix metalloproteases (Mmps) in vSMCs. However, AngII‐induced development of AAA as determined by maximal aortic diameter or increase in blood pressure was not suppressed by AT2R activation.

## 2 MATERIALS AND METHODS

### 2.1 Animals, design and aneurysmal model

Eight‐week‐old male *Apoe^−/−^* (B6.129P2, stock no. 002052) mice were purchased from the Jackson Laboratory. Male mice were preferred for these studies because of high incidence of AngII‐induced AAA as described.^18^ Mice were kept on a 12‐hour/12‐hour light/dark cycle with standard chow. Aneurysmal studies were performed on these mice by infusing AngII for 28 days using published protocols.^19^, ^20^ Some animals were administered with AT2R agonist NP‐6A4 (2.5 mg/kg/day, subcutaneous) until the end of the study (Supporting Figure S1). NP‐6A4 was a gift from Novopyxis Inc (Boston, MA, United States). This dose of NP‐6A4 was selected based on previous studies in Pulakat laboratory using mice with streptozotocin‐induced diabetes that demonstrated optimal tolerance without any adverse effects (unpublished data). Animals were randomly allocated to AngII infusion, NP‐6A4 treatment or control. For AngII infusion, mice were anaesthetized in a closed chamber with 1%‐2% isoflurane in oxygen for 2 to 5 minutes until immobile. Each mouse was then removed and taped on a heated (37 ± 2°C) procedure board with 1.0 ± 1.5% isoflurane administered via nosecone during minor surgery. Mini‐osmotic pumps (Model 2004; ALZET, Cupertino, CA, USA) containing AngII (1 µg/kg/min; Sigma) were implanted subcutaneously in the neck region of anaesthetized mice. At the end of the study, mice were terminated with an overdose of anaesthetics ketamine (150‐200 mg/kg) and xylazine (20 mg/kg). Some of the aortae were dissected, fixed in 10% formalin and processed for macroscopic and histologic studies. Other aortae were processed for gene expression and protein analysis. All the animal‐related experiments were approved by the Animal Care and Use Committee at the University of Missouri (Columbia, MO, USA). The Animal Research: Reporting of In Vivo Experiments (ARRIVE) guidelines were followed in the animal studies.^21^

### 2.2 Transabdominal ultrasound imaging and quantification of aortic aneurysms

Mice were put into the anaesthesia chamber, followed by anaesthetization with oxygen and vaporized isoflurane (~2%), and ultrasound imaging was performed as described previously.^19^, ^22^ Briefly, warmed ultrasound gel was applied to the abdominal surface, and 40‐MHz ultrasound transducer (Vevo MS550D, Toronto, ON) was used to collect B‐mode, M‐mode and ECG‐based kilohertz visualization (EKV) mode images by the imaging system (Vevo 2100, VisualSonics, Toronto, ON) as described.^19^, ^22^, ^23^, ^24^ Briefly, short‐ and long‐axis scans were performed on the abdominal aorta from the level of the left renal arterial branch through to the suprarenal region. Cine loops of 100 frames were acquired throughout the renal region to determine the maximal diameters of the abdominal aorta in the suprarenal region. All the ultrasound data were collected in a blinded fashion by an experienced faculty member in the core facility at Dalton Cardiovascular Research Center for consistency. The AngII‐induced AAA was defined as having at least 50% increase in the maximal intraluminal diameters (MILDs) of the abdominal aorta compared with the control mice in the long axis of M‐mode images obtained from ultrasound. For quantification of the maximal external aortic diameters (MEADs), suprarenal abdominal aortic diameters were measured using ZEN lite software (Zen 2.3 Blue Edition, Zeiss; NY) by an independent researcher ex vivo under a microscope.^22^ MILD and MEAD were measured to depict the in vivo and ex vivo measurements of the diameter of the aorta, respectively, to complement the findings as described previously.^22^ Mice were closely monitored for acute rupture incidences for first 10 days of AngII infusion. The dead mice immediately underwent autopsy, and rupture was defined by the presence of blood clot in the chest cavity and haemorrhage of abdominal aorta between the celiac artery and the left renal artery.

### 2.3 Blood pressure measurement

Blood pressure was measured non‐invasively on conscious mice using a CODA volume pressure recording tail‐cuff system (Kent Scientific Corporation, Torrington, CT) as described previously.^19^, ^20^ Briefly, mice were acclimated for two days to restraint tubes and trial measurements. On the third day, following 5 acclimation cycles, 25 individual blood pressure measurements (technical replicates) were taken; all false readings (as determined by the diagnostic software) were excluded, and any animal failing to register at least 20 (80%) ‘true’ readings was excluded from analysis. Data were trimmed to exclude the lowest and highest 5% of measured values, and the mean was used to represent each animal. The measurement of blood pressure was performed blindly with respect to experimental groups.

### 2.4 AAA classification

AAA complexity was determined by Daugherty's classification by measurement of the aortic diameter and histological features.^25^, ^26^ Type I represents a small single dilation (1.5‐2.0 times of a normal diameter); type II denotes a large single dilation (>2 times of a normal diameter); type III is multiple dilations; and type IV is aortic rupture that leads to death because of bleeding into the peritoneal cavity.

### 2.5 Aortic stiffness, distensibility and radial strain measurement

In vivo aortic stiffness was measured locally in the abdominal aorta by pulse‐wave velocity (PWV) technique by analysing EKV data collected at day 28 of AngII infusion using Vevo Vasc software as described previously.^19^, ^23^, ^27^, ^28^ Vevo Vasc software was used to calculate PWV as a ratio of the distance (d) between two locations along the aorta and time delay (*∆*t) of the pulse wave between both locations and is expressed in m/s. Similarly, EKV data were analysed with the Vevo Vasc software to calculate distensibility and radial strains along the two locations of suprarenal abdominal aorta. The measurements for all PWV, distensibility and radial strains were conducted blindly to the study groups. The measurement of in vitro endothelial cell stiffness was determined in the human aortic endothelial cells (hAOECs; from Cell Applications; passage 3‐passage 5) by atomic force microscopy (AFM) as described.^19^ The cells were pre‐treated with NP‐6A4 (5 μM) for 72 hours with daily NP‐6A4 replenishment. During the final 24‐hour incubation, cells were treated with TNF‐α (10 ng/mL) in the presence or absence of NP‐6A4. Endothelial cell stiffness in the cells was measured using a standard protocol as described.^29^, ^30^

### 2.6 Histology and immunohistochemistry (IHC)

After fixation, the abdominal aortae from experimental mice were rinsed with PBS and processed for paraffin embedding. Serial sections (5 μm) were prepared by cutting abdominal aorta into two equal halves and sectioned throughout the tissue as described.^22^ The sections of the abdominal aorta at regular intervals (200 μm) were subjected to haematoxylin and eosin (H&E), elastin and picrosirius red stain for histoarchitectural evaluation of aneurysm as described previously.^19^ For IHC, the abdominal aortae were stained with antibodies for F4/80 (1:200, ab100790), CD31 (1:300; ab28364), eNOS (1:300; 32027S, Cell Signaling, Danvers, MA), osteopontin (1:1600; ab8448), cleaved caspase 3 (1:200; 9661S, Cell Signaling), Mcp1 (1:400; ab7202) and AT2R (1:200; AAR‐012, Alomone Labs, Jerusalem BioPark, Jerusalem, Israel) as described.^31^ The specificity of all the antibodies was confirmed using appropriate IgG controls (I‐2000‐1, I‐4000‐1, I‐1000‐5; Vector Laboratories, Burlingame, CA) in place of primary antibodies at the same concentrations. The intensity of the immunostaining was evaluated by obtaining 6 images from random areas of interest at 40X from each tissue (n = 6) and quantified using Fiji version of Image J following the software directions as described.^22^, ^32^ Briefly, after opening the representative image in FIJI version of Image J, the appropriate region of interest was outlined with freehand selections. After applying colour deconvolution module, measurements were made on the appropriate image (H‐DAB). The intensity was normalized to the control aortae and shown as arbitrary units. The images were blinded in order to reduce bias during quantification.

### 2.7 Cell isolation, RNA extraction and quantitative real‐time PCR

*Bone marrow–derived macrophages* (BMDMs) were isolated from eight‐week‐old experimental mice treated with AngII or with NP‐6A4 and AngII by previously established protocols.^19^, ^20^, ^31^ Total RNA from cells and aortic tissues was extracted using the RNeasy Mini Kit and RNeasy Fibrous Tissue Mini Kit (Qiagen, Germantown, MD), respectively, following the manufacturers’ instructions. Quantitative real‐time PCR (qPCR) was performed on CFX connect^™^ Real‐time PCR Detection System (Bio‐Rad) in triplicate. Fold change in gene expression was determined by normalizing cycle threshold (CT) values against housekeeping gene (*Rpl13a*). The primer sequences for genes are listed in Supporting Table S1.

### 2.8 Western blotting

Human umbilical vein endothelial cells (hUVECs; from GIBCO, Invitrogen Cell Culture; passage 3‐passage 7) were pre‐treated with NP‐6A4 (5 μM) for 72 hours with daily media change and NP‐6A4 replenishment. Some cells were exposed to TNF‐α (10 ng/mL) alone or in combination with NP‐6A4 for 24 hours before harvesting cells for protein extraction. Cell lysates were prepared, and 20 μg of protein was loaded on SDS‐PAGE gel, electrophoresed and transferred to polyvinylidene fluoride membranes as described.^19^ The primary antibodies used were eNOS (9586; 1:2000), phospho‐eNOS (9575; 1:2000) and GAPDH (NB300‐221; 1:5000). After washing and incubation with appropriate peroxidase‐labelled secondary antibodies for 1 hour, the bands were detected using ECL reagent and the ChemiDoc imaging system. Three independent experiments were performed to ensure the reproducibility of Western blotting data and for quantification.

### 2.9 Nitric oxide (NO) staining

Unfixed fresh aortic rings (1‐2 mm) from AngII and NP‐6A4 plus AngII‐treated *Apoe^−/−^* mice at day 28 were incubated with membrane‐permeable dye 4‐amino‐5‐methylamino‐2′,7′‐difluorofluorescein diacetate (DAF‐FM; 10 μM; Invitrogen, Carlsbad, CA) for 1 hours at room temperature (RT) as described.^33^ After washing two times with fresh HBSS buffer, the aortic rings were immediately snap‐frozen with OCT embedding compound in liquid nitrogen. Frozen rings were then cut into 10‐µm sections and imaged by Lionheart automated microscope. The fluorescent intensity was quantified using Gen 5 software (BioTek, Winooski, VT) as described.^22^

### 2.10 Flow cytometry for NO staining in hAOECs

hAOECs were pre‐treated with NP‐6A4 (5 μM) for 72 hours with daily NP‐6A4 replenishment, washed and incubated with 5 μM DAF‐FM for 30 minutes at RT in the dark. After washing three times in the FACS buffer, the cells were trypsinized and analysed to measure NO level by flow cytometry (BD LSRFortessa X‐20 Instrument, Franklin Lakes, NJ). Live cells were analysed to measure the fluorescence intensity.

### 2.11 Gelatine zymography (GZ) and in situ zymography (ISZ)

For GZ, the aortic tissue lysates from experimental mice with AngII infusions were used as described.^19^ For ISZ, aortic tissues were cut and incubated with substrate solution containing DQ gelatine (D12054; Invitrogen) and ISZ was performed as described.^34^ Negative control sections were treated without DQ gelatine. Sections were mounted with VECTASHIELD medium with DAPI (H‐1800; Vector Labs). Fluorescence intensity in the medial layer of the tissue sections was quantified using Gen 5 software (BioTek).

### 2.12 Statistical analysis

Statistical analyses were performed with GraphPad Prism version 7.0 (GraphPad Software, Inc, San Diego, CA, USA). All the data were assessed for normality and equal variance using Shapiro‐Wilk test and Levene's test, respectively. Unpaired two‐tailed Student's *t* test was used to determine statistical difference between two groups for normally distributed continuous variables. For comparison of multiple groups, ANOVA followed by Tukey's multiple comparison analysis was used. Normally distributed data were analysed by non‐parametric Mann‐Whitney test or Kruskal‐Wallis test. For survival graphs and incidence of AAA, log‐rank test and Fisher's exact test were applied, respectively. Data are presented as median ± interquartile range for the PWV, MILD, MEAD, distensibility and radial strain. For rest of the quantitation, mean ± SEM was calculated. *P* < 0.05 was considered statistically significant for all tests.

## 3 RESULTS

### 3.1 NP‐6A4, an AT2R agonist, stabilizes AngII‐induced aortic stiffness

**Figure 1:**
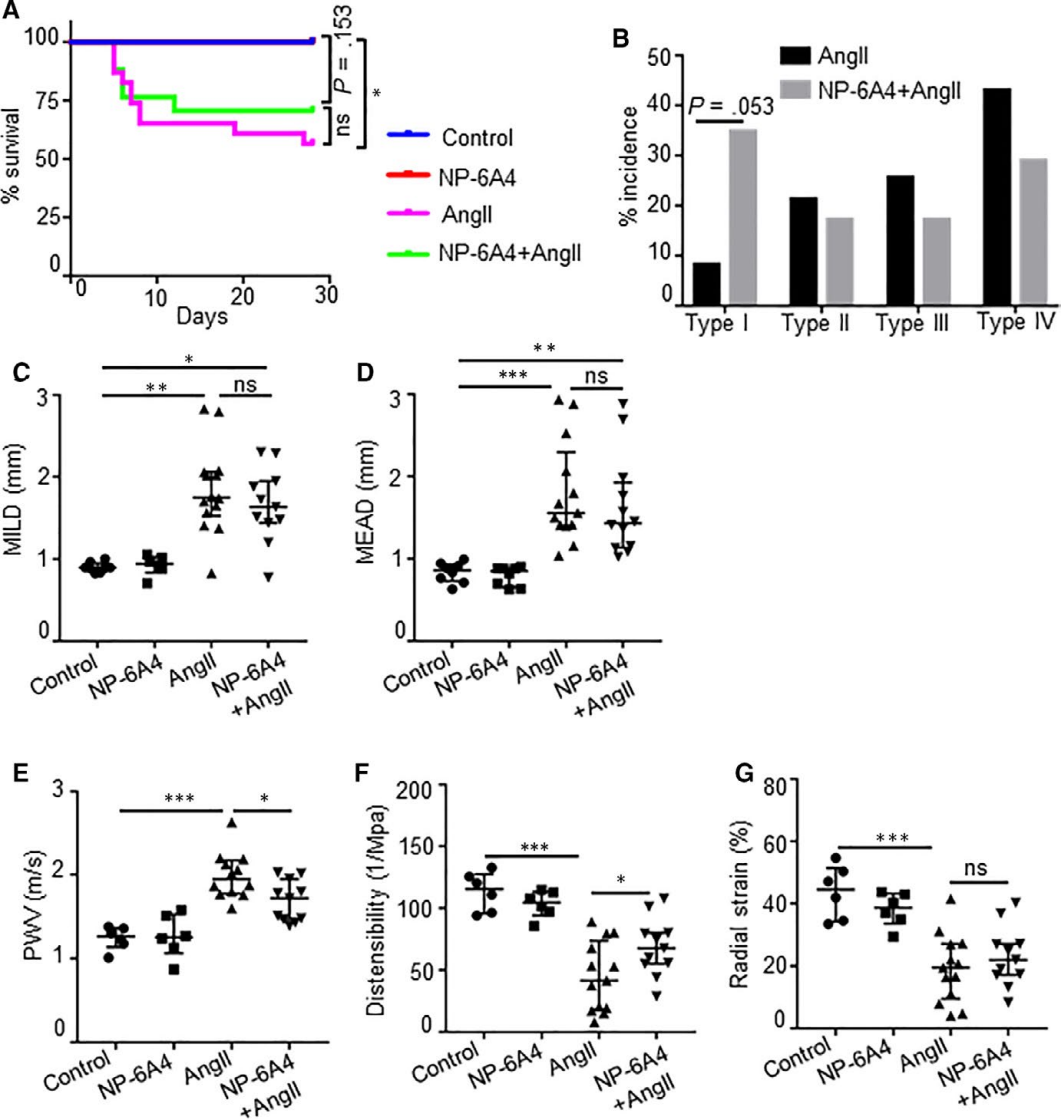
NP‐6A4 attenuates AngII‐induced aortic stiffness in *Apoe^−/−^* mice. A, Graph showing the survival rate of *Apoe^−/−^* mice in response to NP‐6A4 and AngII. Five out of 17 mice died of abdominal aortic rupture in NP‐6A4*–*treated AngII animals, whereas 10 out of 23 mice died in the AngII group. B, Aneurysm severity (type I to type IV) scored using a classification system at day 28. C, Quantification for maximal intraluminal diameter (MILD) as measured by ultrasound at day 28 of AngII infusion. D, Quantification of ex vivo maximal external aortic diameter (MEAD) of suprarenal aorta (mm) by microscopy at day 28. E, Pulse‐wave velocity (PWV) calculated from measurements of abdominal aortic pulse pressure as determined by EKV in response to AngII at day 28. F and G, Distensibility and radial strain at day 28 of AngII and NP‐6A4 treatments as measured by Vevo Vasc analysis. Log‐rank test was used for comparing survival in A. Fisher's exact test was used for analysis in B. Kruskal‐Wallis test was applied for statistics in C and D. Tukey's multiple comparisons test was used for data analysis in E‐G. *P < 0.05; **P < 0.01; ***P < 0.001, ns = non‐significant

To determine whether NP‐6A4*–*induced AT2R activation mitigated AngII‐induced vascular damage, we compared AngII‐induced vasculopathy in *Apoe^−/−^* mice with or without NP‐6A4 treatment. One cohort of mice were subjected to AngII exposure (1 µg/kg/min in mini‐osmotic pumps) for 28 days. AngII infusion resulted in aortic rupture leading to early death of 43.5% animals (10 out of 23). Another cohort of *Apoe^−/−^* mice were pre‐treated with NP‐6A4 (2.5 mg/kg/day) for 14 days before starting AngII exposure. NP‐6A4 treatment reduced early death events marginally to 29.4% (5 out of 17) (Figure 1A). The overall AAA incidence in the AngII groups was modestly higher (87%, 20 of 23) than that in the NP‐6A4 + AngII groups (70.6%, 12 of 17). Daugherty's classification of the abdominal aortic lesions showed majority of the lesions with AngII infusion were type III and type IV, whereas with NP‐6A4 treatment showed a strong trend (*P* = 0.053) towards type I indicating relatively less severity of aortic lesions (Figure 1B). No change in bodyweight was observed between these groups in response to AngII (data not shown). AngII infusion significantly increased the maximal intraluminal diameter (MILD) similarly in both groups as measured by ultrasound (Figure 1C). The macroscopic examination of the aortae at day 28 demonstrated the similar pattern in the maximal external aortic diameter (MEAD) as MILD (Figure 1D). No change in the MILD and MEAD was observed in the saline and NP‐6A4–treated control mice in the absence of AngII (Figure 1C,D and Supporting Figure S4A,B).

Next, we investigated whether NP‐6A4 treatment mitigated AngII‐induced aortic wall dysfunction and damage. Aortic stiffness, as measured by pulse‐wave velocity (PWV), was significantly higher at day 28 with AngII infusion compared with controls (Figure 1E). NP‐6A4 significantly decreased the aortic stiffness (1.99 ± 0.26 m/s vs 1.68 ± 0.23 m/s, *P* = 0.0278) in these mice. AngII infusion significantly decreased the aortic wall distensibility and radial strain measured at the suprarenal aorta compared with saline controls (Figure 1F,G). NP‐6A4 treatment with AngII infusion significantly improved the distensibility, whereas radial strain was slightly improved. These functional parameters remain unchanged in the control groups with saline or NP‐6A4. AngII infusion alone or with NP‐6A4 significantly increased systolic arterial blood pressure as compared to their respective controls such that no significant differences were observed in these two groups at day 28 (Supporting Figure S2). These results demonstrate that AT2R stimulation with NP‐6A4 mitigates aortic stiffness. We also examined whether NP‐6A4 increases the expression of AT2R in the aortic tissues of a mouse model of angiotensin II (AngII)‐induced AAA. Immunohistochemical staining showed increased expression of AT2R in the adventitial region of abdominal aorta in response to AngII, which was further increased with NP‐6A4 pre‐treatment, without significant change at mRNA level (Supporting Figure S3).

### 3.2 NP‐6A4 treatment modulates AngII–induced changes in collagen location and expression in the aortic tissues of *Apoe^−/−^* mice

**Figure 2:**
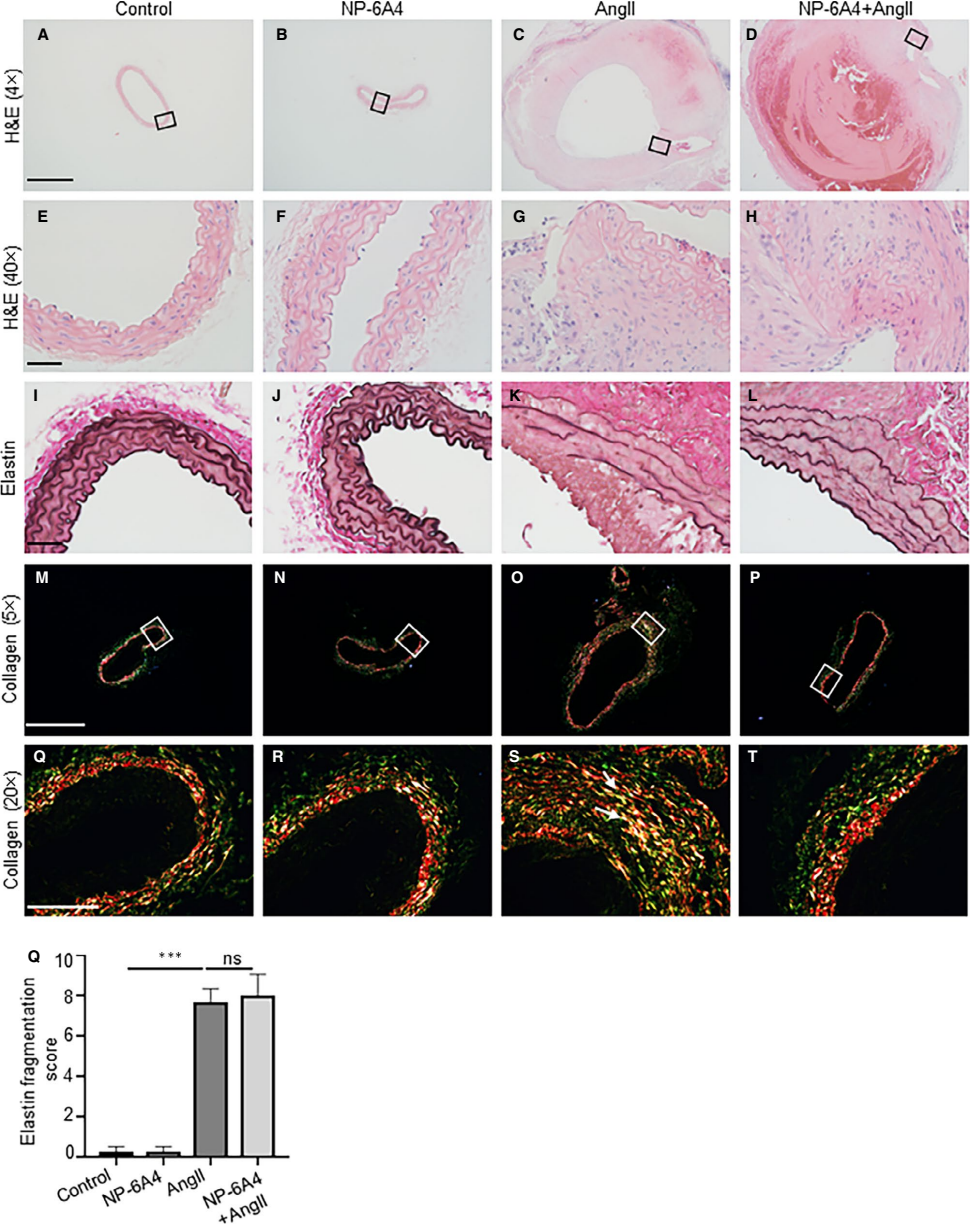
NP‐6A4 modulates AngII‐induced changes in collagen location in the aortic tissues of *Apoe^−/−^* mice. A‐H, Representative images of the transverse sections of aortic sections stained with H&E (haematoxylin and eosin; 4X and 40X), illustrating the extent of aortic thickening at day 28 in indicated experimental groups. I‐L, Representative images of Verhoeff‐van Gieson (VVG) staining demonstrating elastin fragmentation in *Apoe^−/−^* mice infused with AngII at day 28. M‐T, Representative collagen staining using picrosirius red in the experimental groups (n = 6 for each group, 5X and 20X). Q, Quantification of medial elastin degradation in experimental groups. Scale bar = 0.5 mm in A‐D, M‐P and 50 µm in E‐L and Q‐T. ***P < 0.001, ns = non‐significant in Tukey's multiple comparisons test

Aortic tissue cross sections from the experimental mice were subjected to histological analysis to characterize the vascular lesions. No noticeable difference in the adventitial thickening and the cellular infiltrates was observed with or without NP‐6A4 in response to AngII as shown by haematoxylin and eosin (H&E) staining (Figure 2A‐H). A similar extent of elastin degradation was observed with or without NP‐6A4 treatment as determined by Verhoeff‐Van Gieson staining (Figure 2I‐L,Q). However, a closer look at the picrosirius red staining for total collagen content (Figure 2M‐T) demonstrated that in the AngII‐infused mice, increased contents of collagen I (red/yellow fluorescence) collagen III (green fluorescence) were present and particularly localized in the regions of elastin breaks (white arrows, Figure 2S). In NP‐6A4 + AngII‐treated mice, collagen III (green fluorescence) seemed to be minimally expressed (Figure 2T). Additionally, IHC staining of the experimental tissues with antibody specific to collagen I showed decreased staining in the adventitial region of NP‐6A4**–**treated mice (Supporting Figure S5). These data showed that the protective effects of NP‐6A4 on aortic stiffness may partly be associated with NP‐6A4*–*mediated modulation of collagen turnover with the AngII treatment.

### 3.3 NP‐6A4 affects phenotypic changes in aortic tissues

**Figure 3:**
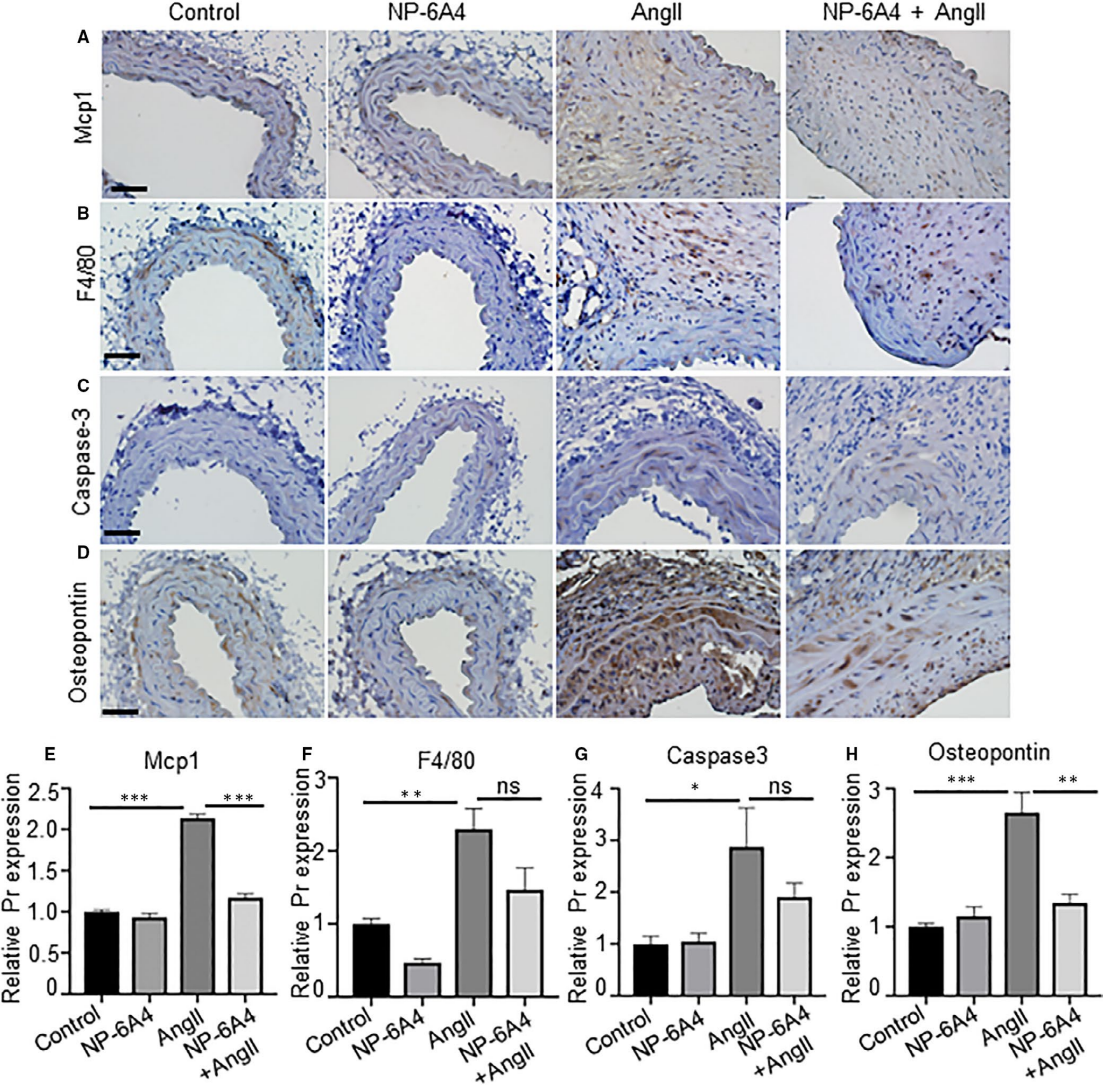
NP‐6A4 affects phenotypic changes in aortic tissues. A‐D, Immunohistochemistry (IHC) images showing expression of Mcp1, F4/80, caspase‐3 and osteopontin in the aortic tissue at day 28 of experimental *Apoe^−/−^* mice. E‐H, Quantification data of Mcp1, F4/80, caspase‐3 and osteopontin expressions in the aortic tissue of indicated mice. Scale bar = 50 µm. *P < 0.05, **P < 0.01, ***P < 0.001 in Tukey's multiple comparisons test (E‐H)

Monocyte chemoattractant protein‐1 (MCP1), a chemotactic cytokine, is reportedly regulated by AT2Rs.^35^, ^36^ Therefore, we examined whether NP‐6A4‐AT2R signalling affects Mcp1 expression of aortic tissues in mice exposed to AngII. As shown in Figure 3A,E, a significant increase in Mcp1 expression in AngII‐treated mice was observed, which was diminished in mice treated with NP‐6A4. In contrast, expression of F4/80, a marker for macrophage contents, was not significantly decreased with NP‐6A4 treatment in response to AngII (Figure 3B,F). In addition, no change in the mRNA expression of inflammatory genes (*iNos*, *Il6* and *Tnfα*) was observed in macrophages isolated from experimental mice (Supporting Figure S6). Next, to understand whether NP‐6A4 treatment modulated apoptosis, we examined expression of active caspase‐3 in these tissues. Active caspase‐3 immunostaining increased significantly in the aorta of *Apoe^−/−^* mice in response to AngII. In the NP‐6A4 + AngII‐treated mice, no significant decrease in the active caspase‐3 immunostaining was observed (Figure 3C,G). Overall, these data suggest that the effects of NP‐6A4 on inflammatory response and apoptosis are minimal and may not contribute to the decreased aortic stiffness observed in these experimental mice.

### 3.4 NP‐6A4 treatment suppresses AngII‐induced osteopontin and proteolytic activity

**Figure 4:**
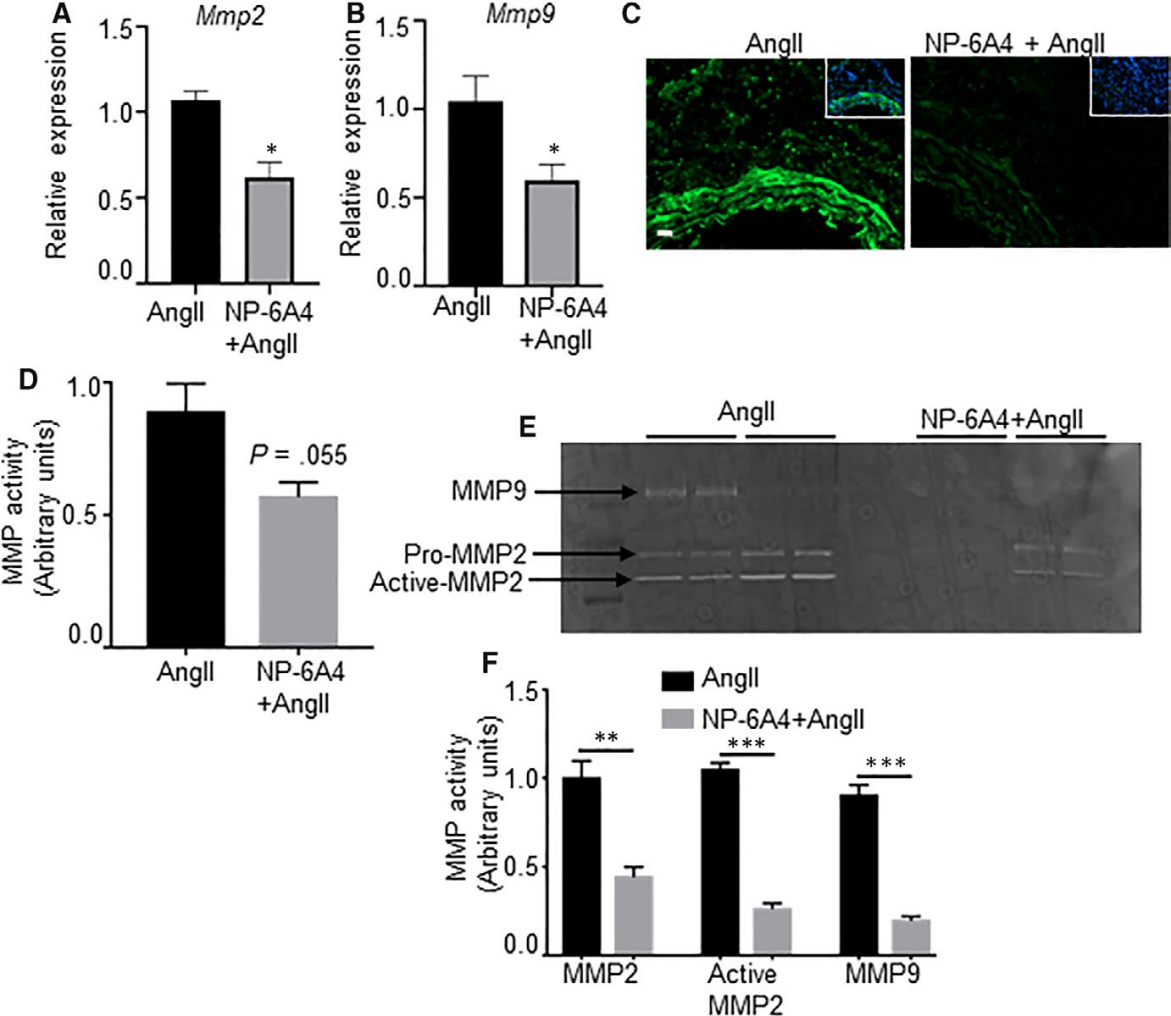
Proteolytic activity is attenuated with NP‐6A4 in AngII‐induced *Apoe^−/−^* mice. A and B, mRNA expression of Mmp2 and Mmp9 in abdominal aortae from *Apoe^−/−^* mice treated with AngII alone or NP‐6A4 and AngII as measured by qRT‐PCR (n = 4 mice per group). C and D, Detection and quantification of gelatinolytic activity of matrix metalloproteinase (MMP) by in situ zymography (ISZ) (n = 4). Green and blue represent MMP activity and nuclear staining of cells by DAPI, respectively. E and F, Representative gelatine zymography and quantification for pro‐matrix 2 (MMP2), active MMP2 and MMP9 in aortic tissue lysates from *Apoe^−/−^* mice with indicated treatment groups (n = 4 mice per group). Pr = protein. Scale bar = 50 µm. *P < 0.05, **P < 0.01, ***P < 0.001 in paired two‐tailed Student's *t* test

Next, we examined the expression of osteopontin (OPN), a matrix protein that modulates SMC proliferation and apoptosis.^37^ Specifically, matrix metalloproteinases (MMP)‐2 and MMP‐9 are OPN‐dependent molecules that promote vascular instability.^38^ Increased expression of OPN was observed in the adventitial layer of mice aorta exposed to AngII. This AngII‐induced increase in OPN was suppressed by NP‐6A4 treatment (Figure 3D,H). A significant reduction in *Mmp2* and *Mmp9* gene expression was observed with NP6‐A4 treatment in the abdominal aortae of experimental mice (Figure 4A,B). We further confirmed the NP‐6A4**–**mediated reduction in MMP activity in the aortic tissues by in situ zymography and gelatine zymography (Figure 4C‐F). These data suggest that NP‐6A4 may attenuate aortic stiffness via decreasing MMP‐mediated proteolytic activity.

### 3.5 NP‐6A4 attenuates endothelial dysfunction

**Figure 5:**
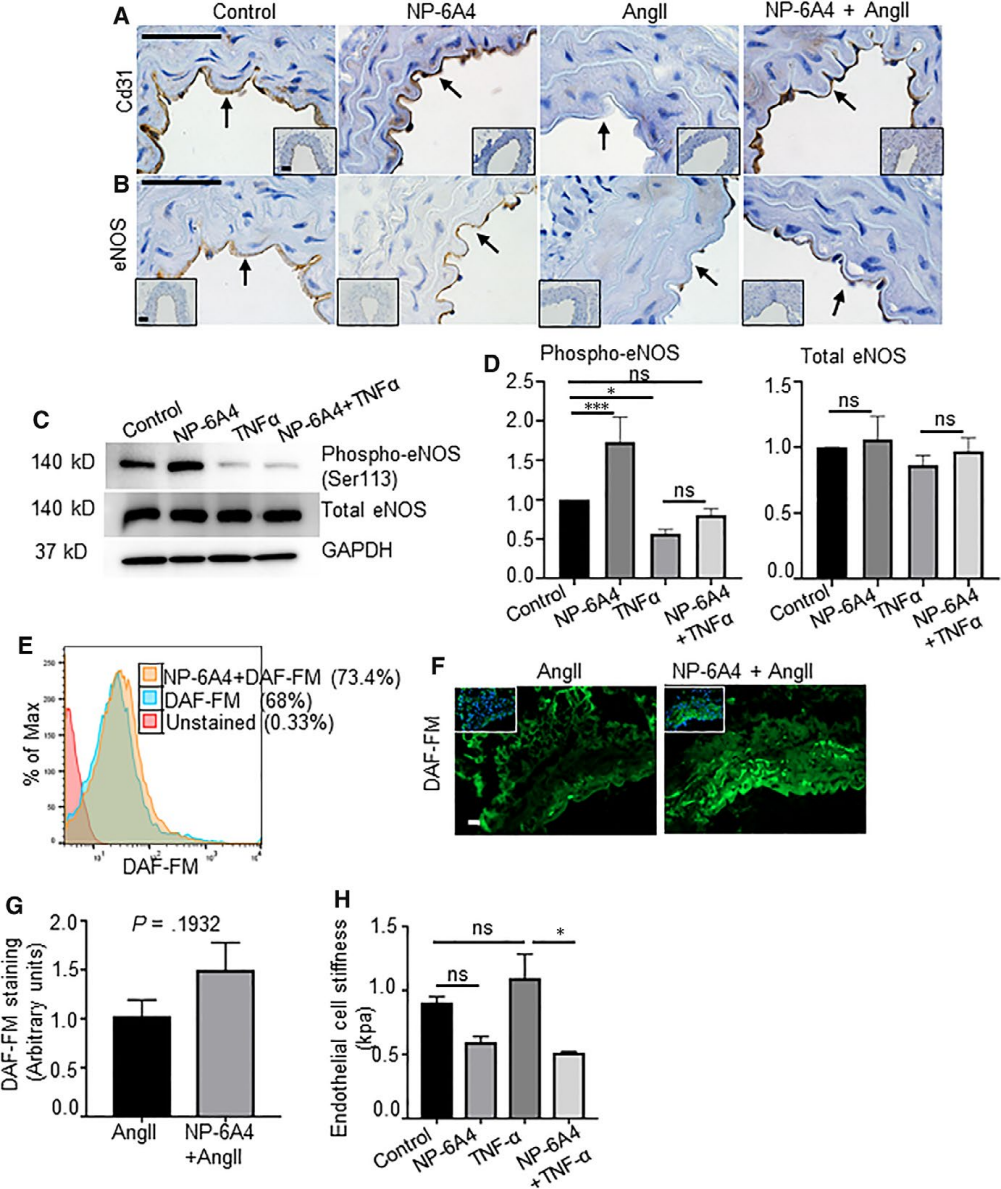
NP‐6A4 improves endothelial function and attenuates aortic stiffness. A and B, IHC images showing expression of Cd31 and eNOS in the aortic tissue at day 28 of experimental *Apoe^−/−^* mice. C and D, Western blot and its quantification showing expression of phospho‐eNOS, total eNOS and GAPDH in the human umbilical vein endothelial cells (hUVECs) with various treatment. E, Representative flow cytometry histogram measuring nitric oxide (NO) production in the human aortic endothelial cells (hAOECs) pre‐treated with NP‐6A4 using DAF‐FM staining. F and G, Representative ISZ image for measuring NO production using DAF‐FM staining in the aortic tissues from experimental *Apoe^−/−^* mice at day 28 of AngII and its quantification. H, Endothelial cell stiffness in the hAOECs with indicated treatment groups measured by atomic force microscopy (AFM). Scale bar = 50 µm. *P < 0.05, ***P < 0.001 in paired two‐tailed Student's *t* test

Endothelial cells (ECs) play a critical role in vascular stiffness by maintaining bioavailability of NO and proper vasodilation. We previously reported that AT2R activation with NP‐6A4 increases NO bioavailability in human coronary artery endothelial cells.^8^ We first examined the CD31 and eNOS expression in the aortic vascular wall of experimental *Apoe^−/−^* mice. AngII infusion markedly decreased the CD31 and eNOS expression in the endothelial cell‐enriched intimal layer of the vascular wall (Figure 5A,B). NP‐6A4 treatment reversed this AngII‐induced effect on endothelial layer (Figure 5A,B). Both CD31 and eNOS expressions were similar in the saline control and the NP‐6A4–alone groups.

To delineate the mechanistic role of NP‐6A4 on the ECs, we investigated the expression and phosphorylation levels of eNOS and NO generation in hUVECs and hAOECs in the presence of TNF‐α (10 ng/mL), with and without exposure to 5 μM NP‐6A4. As shown in Figure 5C,D, phospho‐eNOS levels were significantly increased by NP‐6A4 at the basal level. TNF‐α significantly suppressed the eNOS phosphorylation. However, pre‐treatment of hUVECs with NP‐6A4 partially rescued the TNF‐α*–*mediated suppression of phospho‐eNOS. In addition, DAF‐FM staining of hAOECs by flow cytometry revealed increased fluorescence with NP‐6A4 treatment suggesting enhanced NO production with NP‐6A4 at the basal level (Figure 5E). Fresh aortic tissue sections also demonstrated slightly increased NO production in response to NP‐6A4 compared with AngII controls (*P* = 0.1932) (Figure 5F,G). Importantly, AFM of hAOECs treated with TNF‐α in the presence or absence of NP‐6A4 revealed significant attenuation of endothelial cell stiffness with NP‐6A4 treatment (Figure 5H). Collectively, these data suggest that AT2R activation by NP‐6A4 is upstream of NO release and is crucial for decreasing endothelial cell*–*mediated aortic stiffness. Moreover, since NO is known to regulate MMP activity,^39^ these data suggest that NP‐6A4 may decrease proteolytic activity possibly via regulation of NO.

## 4 DISCUSSION

AngII‐induced vasculopathies, particularly AAA, has been studied extensively in male mice. Although AngII binds to both AT1R and AT2R in the vascular cells, the AngII‐AT1R*–*induced inflammation overrides any protection induced by activation of AT2R by AngII in these models. Importantly, whether specific activation of AT2R by an agonist would mitigate AngII‐induced vasculopathies such as AAA is not known. Here, we investigated whether vascular protective effects of NP‐6A4‐AT2R signalling on vasculopathies induced in male *Apoe* ^‐/‐^ mice via AngII (1 µg/kg/day) infusion. Results presented here show that NP‐6A4, a peptide agonist of the AT2R, exerts specific anti‐inflammatory and vascular protective effects, but does not change AngII‐induced increase in blood pressure or size of AAA. Specifically, NP‐6A4‐AT2R signalling was effective in attenuating AngII‐AT1R*–*induced endothelial cell stiffness that underlies the development of vascular pathologies. Mechanistically, NP‐6A4‐AT2R signalling improved aortic distensibility via increasing bioavailability of NO and inhibiting the osteopontin‐matrix metalloprotease pathway.

The partial effects of NP‐6A4 on AAA can be attributed to several factors in this study. (a) We used to a relatively lower dose of NP‐6A4 for a longer duration to minimize adverse effects. It will be interesting to examine whether higher dose of NP‐6A4 (up to 20 mg/kg/day can be tolerable to mice without any side‐effects; unpublished data) extends its protective role to luminal diameter and inflammatory response. (b) In the present study, we examined prophylactic effects of NP‐6A4 on AAA. Our recent findings indicate that aortic stiffness rather than size of AAA is a determinant factor of stability in the small/actively growing AAAs.^22^ Hence, it is highly conceivable that attenuating aortic stiffness and improving distensibility of the aorta with NP‐6A4 may stabilize the AAAs as therapeutic treatment. (c) The study is also limited by the fact that as we are utilizing a competitive AngII‐induced AAA model, AT1R might be overriding the protective effects of AT2R agonist. We observed a significant reduction in *Agtr1b* (AT1Rb subtype) in response to NP‐6A4, but not in *Agtr1a* (AT1Ra subtype). It is conceivable that reduction in AT1Rb partially contributes to NP‐6A4's protective effects in the aorta; however, further studies are needed to verify this possibility. It will be very intriguing to examine the effects of NP‐6A4 in the elastase or calcium chloride models of aneurysm. Overall, our studies highlight previously unknown protective effects of AT2R agonist on aortic stiffness and proteolytic activity, which may have potential implications to preserve the aortic integrity of the aorta in vascular pathologies.

The formation of AAA includes the degradation of medial extracellular matrix and subsequent medial rupture, followed by complex cellular changes in the intima, media and adventitia.^25^, ^40^ Additionally, the progression and rupture of aneurysms are known to be influenced by aortic stiffness as well as the blood pressure (BP). Reduced severity of AAA observed in response to NP‐6A4 pre‐ and co‐treatments correlated with decreased aortic stiffness, and this result is in agreement with the previous report.^14^ Moreover, the effect of NP‐6A4 treatment on the collagen content and location in the aortic tissues from mice with AngII‐induced AAA suggests that protective effect of NP‐6A4 on AngII‐induced vascular pathology is in part mediated via attenuating collagen disorganization. We speculate that there is a higher turnover of the collagens in the aorta because of increased proteolytic activity in response to AngII and NP‐6A4 co‐treatment that suppresses proteolytic activity of MMPs reduces collagen degradation and improves vascular stability.

AngII treatment increased BP in *Apoe* ^‐/‐^ mice, but NP‐6A4 did not suppress this effect. Previous reports show that systemic administration of AT2R agonist C21 did not significantly change BP,^41^ indicating that systemic administration of AT2R agonists does not cause BP‐lowering effect. Thus, the protective effects of NP‐6A4 on AngII‐induced aortic pathology are not the direct effect of inhibition of AngII‐AT1R*–*induced increase in BP. Moreover, it is largely known that aortic inflammation and proteolysis promote AAA in AngII‐induced mouse models and are not because of the secondary increase in BP.^17^, ^20^, ^40^ Based on our observation that NP‐6A4 co‐treatment suppressed expression of osteopontin as well as expression and proteolytic activity of MMP2 and MMP9, we propose that protective effects of NP‐6A4 on AngII‐induced aortic pathology are mediated via suppression of osteopontin‐MMP2‐MMP9 pathway.

NP‐6A4 is an AT2R selective agonist and does not compete with AngII to bind the AT1 receptor. We have recently shown that NP‐6A4 increases expression of *Agtr2* mRNA and AT2R protein in human cardiovascular cells and has protective effects via increased cellular respiration, eNOS signalling and higher level of NO.^5^, ^8^ AT2 receptors are expressed on ECs, vSMCs and immune cells.^8^ Importantly, the expression of Cd31 and eNOS levels was augmented in response to NP‐6A4 treatment in the aortic tissues of mice with AngII‐induced AAA. The endothelial dysfunction contributes to AAA development because of increased oxidative stress mediated by impaired NO bioavailability.^42^ In addition, endothelial dysfunctions also influence arterial remodelling by causing phenotypic changes in the vSMC‐enriched medial layer including proliferation, migration and apoptosis.^43^, ^44^ it is conceivable that NP‐6A4‐AT2R**–**induced increase in phospho‐eNOS and bioavailability of NO could have improved endothelial function and thus mitigated AngII‐induced aortic stiffness.^8^ Although AT2R stimulation is shown to be anti‐inflammatory in macrophages,^45^ our data indicate that decreased Mcp1 and proteolytic activity observed in our model could be contributed from cell types other than macrophages such as CD4^+^ T cells. However, it is also true that inflammation plays an active role in the progression of AAA.^25^, ^31^ In the future, it will be interesting to dissect the protective cell types associated with NP‐6A4*–*mediated protection in the AngII‐induced mouse model of AAA.

In conclusion, AT2R stimulation with NP‐6A4 attenuated only aortic stiffness in response to AngII in mouse model of AAA. Increased levels of phospho‐eNOS and NO availability that can result in restoration of endothelial dysfunction, and inhibition of proteolytic activity were the major pathways found to be involved in protective effects of NP‐6A4. Increased RAS activity and elevation of AngII are one of the mechanisms for the development of AAA. As AngII can activate AT2R, the fact that AngII infusion causes AAA in male mice indicates that AngII‐induced AT2R activation that can happen at the same time as AngII‐induced AT1R activation in male aortic tissue is not sufficient to prevent development of AAA. Conversely, female mice that express higher levels of AT2R in their vasculature are better protected from AngII‐induced AAA.^46^ Importantly, our data show that NP‐6A4‐AT2R signalling mitigates AngII‐induced aortic stiffness in male mice suggesting that NP‐6A4 treatment could be effective in restoring vascular protective AT2R signalling in male mice that have less AT2R expression compared with their female counterparts. Thus, this study can be of clinical significance because of potential implication of this AT2R agonist in the treatment options for AAA, a male predominant disease.

## CONFLICT OF INTEREST

The authors confirm that there are no conflicts of interest.

## AUTHOR CONTRIBUTIONS

NS, LP and CPH conceived and designed experiments and interpreted data. NS, AM, RT and CPH performed experiments. NS and CPH analysed the data, compiled the figures and wrote the manuscript. NS, CPH and LP edited the manuscript.

## Supporting information

Supplementary Material

## Data Availability

All data are available in the manuscript. The data that support the findings of this study are available on request from the corresponding authors.
