# Supplementary material for: AT2R agonist NP‐6A4 mitigates aortic stiffness and proteolytic activity in mouse model of aneurysm
Source: J Cell Mol Med. 2020 May 18;24(13):7393–404. doi: 10.1111/jcmm.15342 (PMC7339180; doi:10.1111/jcmm.15342)
Supplement: Supplementary file 1 — Supplementary Material [file JCMM-24-7393-s001.pdf]

Supplemental Table 1: List of mouse primer sequences used for qRT-PCR studies

Gene	Forward Primer (5'-3')	Reverse Primer (5'-3')
Il6	CTA CCC CAA TTT CCA ATG CT	ACC ACA GTG AGG AAT GTC CA
Mmp2	GAT GTC GCC CCT AAA ACA GA	TGG TGT TCT GGT CAA GGT CA
Mmp9	AGA CCT GAA AAC CTC CAA CCT CAC	TGT TAT GAT GGT CCC ACT TGA GGC
Il10	GCTCTTACTGACTGGCATGAG	CGC AGC TCT AGG AGC ATG TG
Tnfa	CCC ACT CTG ACC CCT TTA CT	TTT GAG TCC TTG ATG GTC GT
iNos	CTC GGA GGT TCA CCT CAC TGT	TCC TGA TCC AAG TGC TGC AGA
At2r	ATG ATT GGC TTT TTG GAC CTGT	AAG GGT AGA TGA CCG ATT GGT
At1ar	GGA AAC AGC TTG GTG GTG AT	ACA TAG GTG ATT GCC GAA GG
At1br	TAC GCC AAG GAA TGA TGA CA	GTC CAC TAC GTC CGC AAT TT

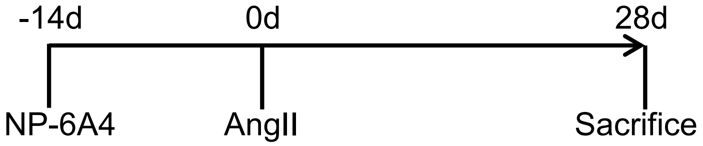

NP-6A4 (**2.5** mg/kg/day) was given subcutaneously in *Apoe*$^{-/-}$ mice on alternate days starting 14 days before AngII infusion and continued for 28 days after AngII.

Supplemental Figure 1. Experimental strategy for *in vivo* studies.

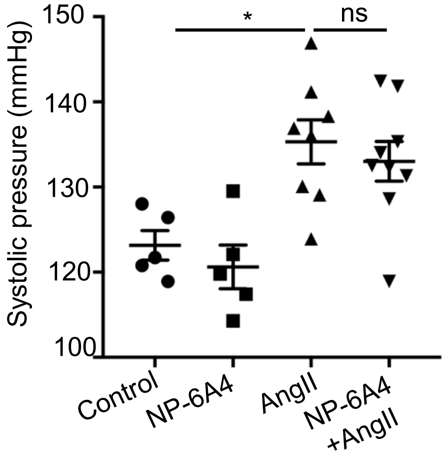

Supplemental Figure 2. NP-6A4 treatment did not affect the systolic arterial blood pressure significantly in *Apoe*$^{-/-}$ mice at baseline or in response to AngII.

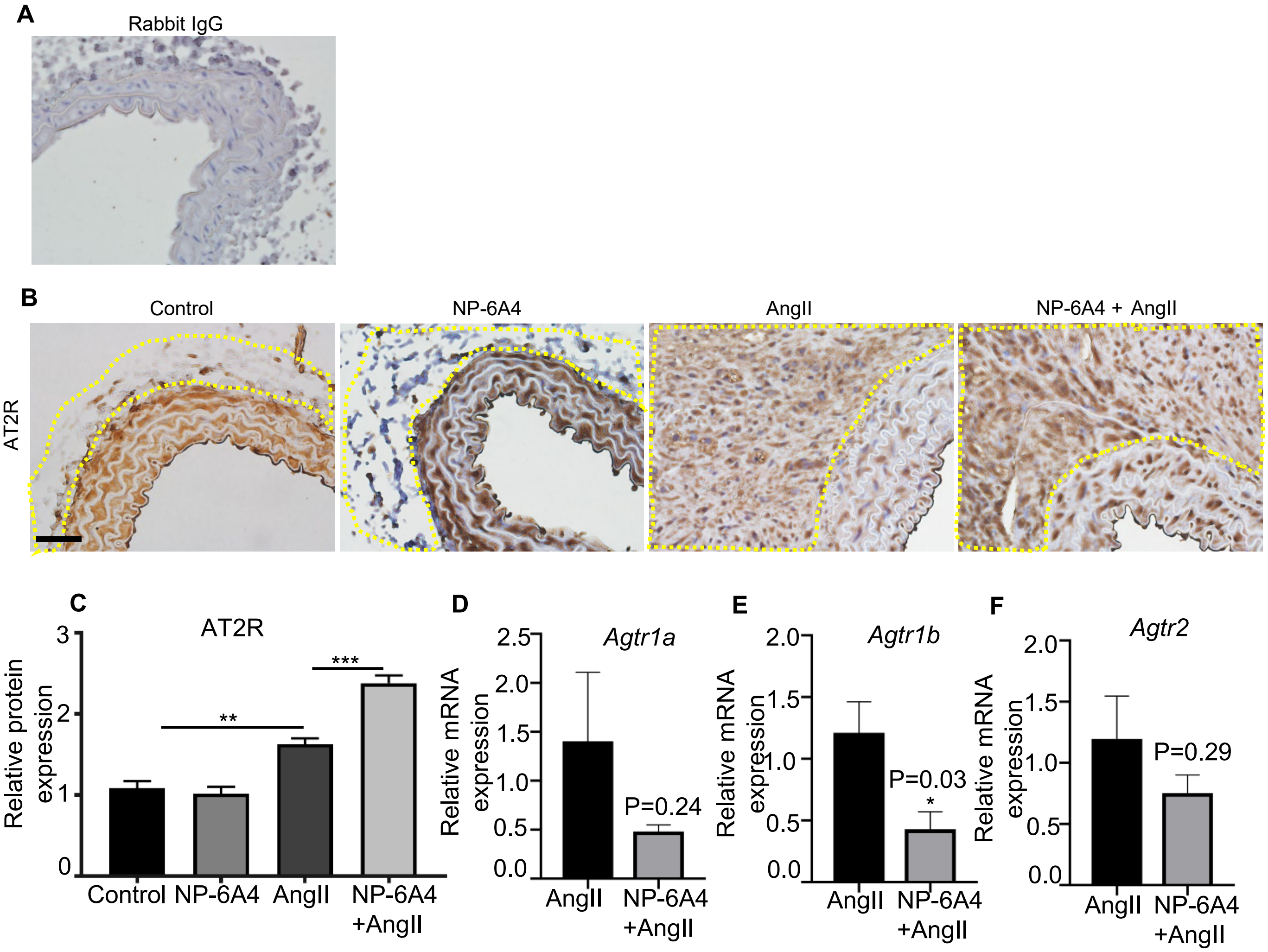

Supplemental Figure 3. Increase in AT2R protein (encoded by *Agtr2* gene) expression in the adventitial region of aorta in response to NP-6A4 treatment and AngII infusion in the $Apoe^{-/-}$ mice. (**A**) Immunohistochemistry (IHC) showing rabbit IgG staining in place of AT2R primary antibody as a negative control staining. (**B**), IHC showing expression of AT2R, NP-6A4 agonist, in the aortic tissue at day 28 of indicated mice (Scale bar = 50 μm). (**C**), Quantification of AT2R protein expression in aortic tissues relative to control. (**D-F**), mRNA expression of mouse *Agtr1a* (AT1a), *Agtr1b* (AT1b) and *Agtr2* in aortic tissues from experimental $Apoe^{-/-}$ mice in the indicated groups as measured by qRT-PCR (n=4 mice in each group). The indicated values are relative to AngII expression level.

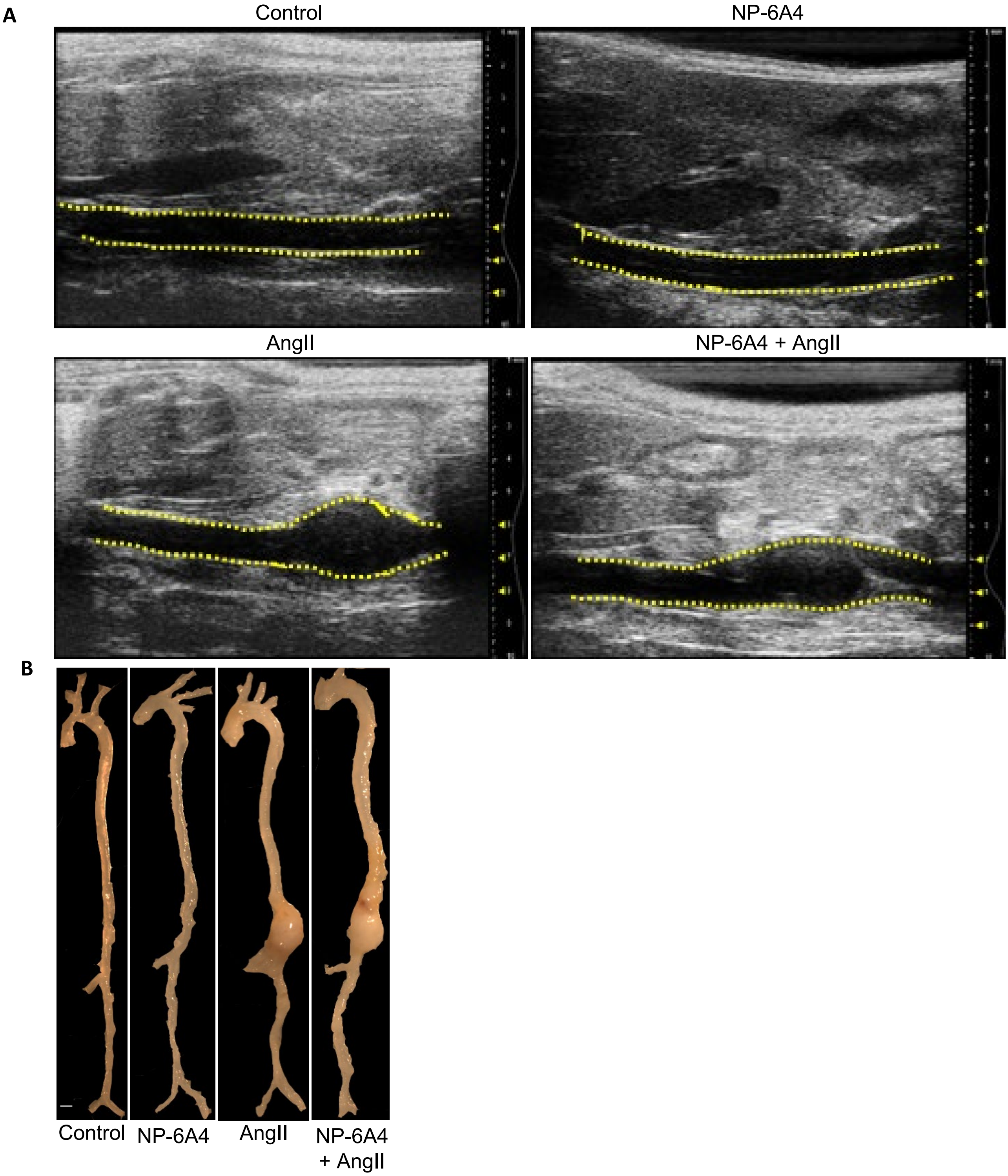

Supplemental Figure 4. (**A**), Representative transabdominal ultrasound images obtained at day 28 using a VisualSonics Vevo2100 showing an increase in the luminal expansion of abdominal aorta in *ApoE*$^{-/-}$ mice in response to AngII. (**B**), Representative aortae from experimental *ApoE*$^{-/-}$ mice showing maximal aortic width in the suprarenal region of the aorta. Images were taken using Zeiss Stemi 2000-C microscope. Yellow dotted lines outline the lumen of the aorta in A. Scale bar, 1mm.

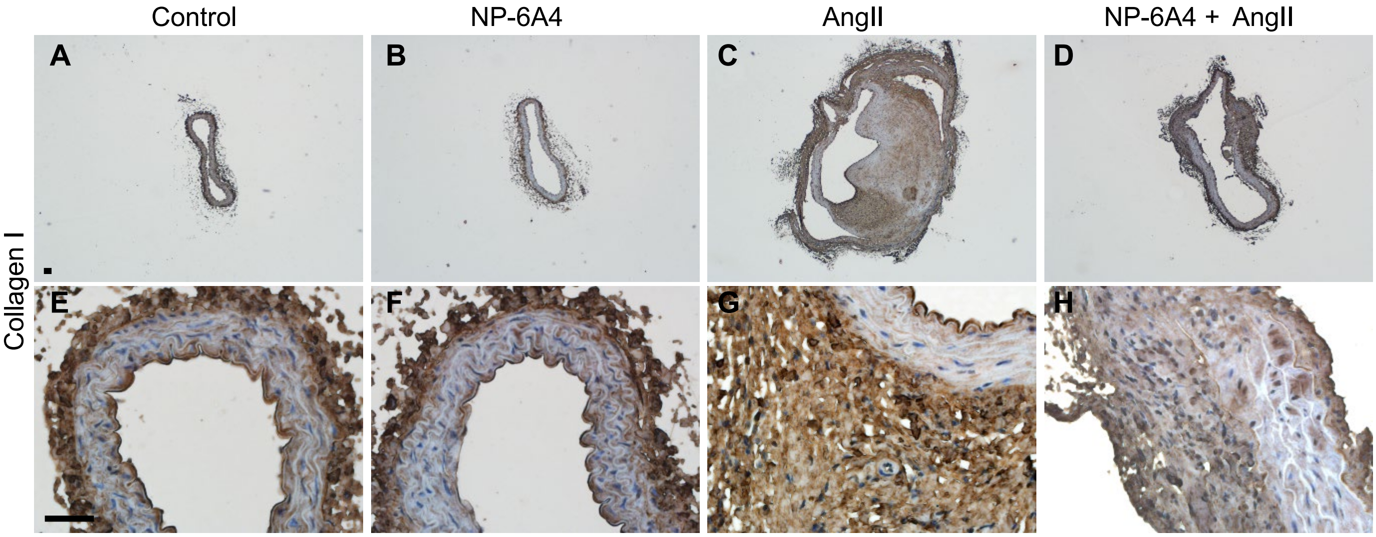

Supplemental Figure 5. (**A** - **H**), Representative IHC images showing expression of collagen I in the aortic tissue at day 28 of experimental *Apoe*$^{-/-}$ mice. 4 X and 40 X images are shown in A-D and E-H, respectively. Scale bar = 50 μm.

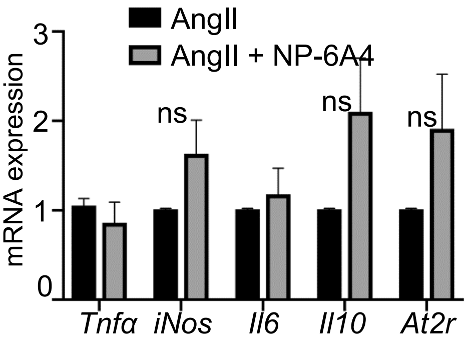

Supplemental Figure 6. mRNA expression of *Tnfα, iNos, Il6, Il10* and *Agtr2* in bone-marrow derived macrophages isolated from mice treated with either AngII alone or with AngII plus NP-6A4 treatments as measured by qRT-PCR. ns=non significant in paired two-tailed Student *t* test.
